# Real-Time Analysis of Laser-Induced Plasmon Tuning in Nanoporous Glass Composite

**DOI:** 10.3390/nano10061131

**Published:** 2020-06-08

**Authors:** Maksim M. Sergeev, Roman A. Zakoldaev, Tatiana E. Itina, Pavel V. Varlamov, Galina K. Kostyuk

**Affiliations:** 1 Faculty of Laser Photonics and Optoelectronics, ITMO University, 197101 Saint Petersburg, Russia; mmsergeev@itmo.ru (M.M.S.); tatiana.itina@univ-st-etienne.fr (T.E.I.); p.v.varlamov@itmo.ru (P.V.V.); gkkostiuk@itmo.ru (G.K.K.); 2 Laboratoire Hubert Curien, UMR CNRS 5516/UJM/Univ. Lyon, Bat. F, 18 rue du Pr. Benoit Lauras, 42000 Saint-Etienne, France

**Keywords:** nanocomposites, porous glass, laser writing, plasmon resonance, nanoparticles, effective medium theory

## Abstract

Laser-induced structuring in nanoporous glass composites is promising for numerous emerging applications in photonics and plasmonics. Local laser irradiation activates an interplay of photo-thermo-chemical mechanisms that are extremely difficult to control. The choice of optimum laser parameters to fabricate structures with desired properties remains extremely challenging. Another challenging issue is the investigation of the properties of laser-induced buried structures. In this paper, we propose a way to control the plasmonic structures formation inside a nanoporous glass composite with doped silver/copper ions that are induced by laser irradiation. Experimental and numerical investigations both demonstrate the capacities of the procedure proving its validity and application potential. In particular, we register transmitted laser power to analyse and control the modification process. Spectral micro-analysis of the irradiated region shows a multilayer plasmonic structure inside the glass composite. Subsequently, the effective medium theory connects the measured spectral data to the numerically estimated size, concentration, and chemical composition of the secondary phase across the initial GC sample and the fabricated structure.

## 1. Introduction

New functional glass composites with embedded nanoparticles are applied in photonics [1,2] in order to fabricate nonlinear light guiding components [3], luminescent [4], and plasmonic [5] structures, bimetallic nanoparticles [6], or colour palettes [7]. Particularly, nanoporous glass with multiple buried hollow channels and pores with well-controlled size represents a promising base for glass composite development [8]. Such a nanoporous framework captures a wide class of dopants: silver halides [9], rare-earth metals [10], or nanoparticles [11]. Laser treatment changes the internal structure of a nanoporous glass composite and tunes its plasmonic properties. However, laser irradiation initiates high-rate photo-thermal-chemical mechanisms [12], which complicate the search for optimal irradiation modes.

Generally, laser irradiation forms a buried modified region in a nanoporous glass composite, more specifically a multilayer spherical plasmonic structure with the concentration of nanoparticles distributed across the cross-section [13]. Moreover, processes, such as growth [14], fragmentation [15], and reduction or oxidation of nanoparticles occur in the irradiated zone, thus defining the structure optical properties that are commonly measured after laser processing by contact examination. However, this approach to measurements remains semi-empirical and cannot explain all of the physical and chemical transformations involved.

Obviously, the real-time investigation of micro-sized buried structures is preferable during laser irradiation. The determination of the optical characteristics, such as refractive index, extinction, and absorption coefficient of the structure, remains challenging. For this, we develop a procedure allowing for one to predict the optical properties of fabricated structures and determine the required laser exposure time and power.

Several optical models, such as Maxwell-Garnett [16] and Burgemann-Bergman [17], were used to simulate optical properties of the glass composite. Additionally, Mie theory and Clausius–Mossotti equation were also used [18]. For simplicity of simulation, the processes of nanoparticle formation, growth, and decay/oxidation were, however, often neglected. Nevertheless, laser irradiation inevitably causes these processes creating additional conditions in the simulation.

Herein, we propose a novel real-time control procedure combining both experimental investigation and simulation to fabricate micro-sized structures inside nanoporous glass doped with silver and copper ions by laser irradiation. In particular, we propose a model that accounts for the time dependent changes in the optical properties during laser irradiation. This procedure requires the registration of the transmitted laser power by power detectors in combination with a careful numerical simulation, which makes the procedure low-cost and easily implementable. For this, we have chosen to simulate plasmonic properties using the effective medium theory (EMT) [19], as this approach is the most relevant under the considered experimental conditions. Experimentally, spectral non-contact measurements are used here to obtain the size, concentration, and chemical composition of the secondary phase across the fabricated structure. Furthermore, physical mechanisms of laser radiation interaction with a nanoporous glass composite are also discussed.

## 2. Materials, Laser Procedure and Modelling

### 2.1. Materials

In the experiment, a plane-parallel glass composite (GC) sample with 1.5 mm thickness is subjected to the near-infrared continuous wave (CW) laser irradiation procedure. GC based on a porous glass (PG) is impregnated with silver/copper halides in the ratio 1:10. Note that the average pores radius, which is around 4 nm and 26% of the total porosity, limits the halides size up to 12 nm [20]. GC chemical composition is the following: 0.25 Na_2_O – 3.42 B_2_O_3_ – 96.09 SiO_2_ – 0.24 Ag_2_O. The sample preparation is a complex and multi-stage procedure, which includes PG fabrication that is based on two-phase alkali borosilicate glass thermal treatment and the following chemical etching to delete a borate phase [21,22]. The final impregnation procedure with silver/copper halides is described elsewhere [23]. Here, we consider a silicate matrix with embedded ions of Na and B in terms of residual trace elements and nanopores filled with Ag/Cu ions.

**Figure 1:**
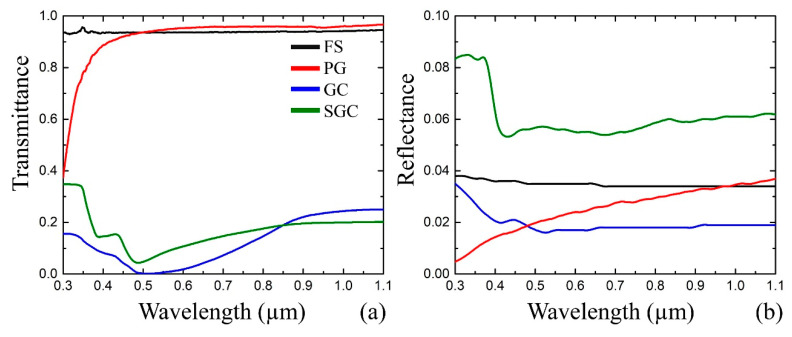
(**a**) Measured transmittance and (**b**) reflectance spectra of porous glass (PG) (red line), glass composite (GC) (blue line), sintered GC (SGC) (green line), and fused silica (FS) (black line) in the range from 0.3 to 1.1 μm.

An additional three samples, such as PG without any dopants, fully sintered GC (SGC) [24], and fused silica (FS), are applied as the reference samples for the simulation stage. Sample transmittance and reflectance are measured in the range from 0.3 to 1.1 μm by a spectrophotometer (MSFU-K Yu-30.54.072, LOMO, St. Petersburg, Russia), where the minimum registration region is equal to 2 µm (Figure 1). The transmittance *T_meas_* and reflectance *R_meas_* of all the samples are measured at normal incidence of light. Spectral curves show that initial PG is similar to FS, except for the UV absorption. Introducing halides in the PG results in a significant absorption in the range from 0.45 to 0.7 µm. As for the laser procedure, the preliminary spectral samples characterization helps to choose the correct laser source wavelength for processing inside the sample. Section 3.4 discusses plasmonic features.

### 2.2. Laser Irradiation

**Figure 2:**
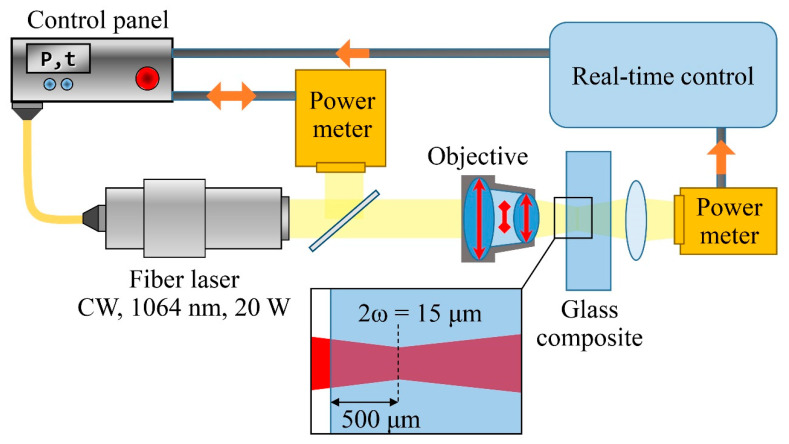
Schematics of the experimental setup for micro-sized plasmonic structures fabrication inside GC.

A commercially available CW fiber laser source is chosen to induce both silica matrix and salt mixture modification triggering local optical changes inside GC. The wavelength (*λ_laser_* = 1064 nm) differs from the maximum absorption of GC (Figure 1a) and enables gentle processing. Figure 2 schematically shows the setup used for the experiment. The CW fiber laser source provides the maximum power *P_0_* = 20 W in the TEM_00_ mode with beam quality *M*^2^ = 2, while the radiation divergence equals 20 mrad and the beam size incident on an objective equals 8 mm. The laser stabilizes within 100 microseconds after turning it on. The objective (10×, NA = 0.25) is used to achieve the laser beam waist diameter (*2ω_0_*) to be equal to 15 µm and its length 40 µm. The beam waist is located at 500 µm below the GC surface. Incident laser power (*P_0_*) and the transmitted one (*P_1_*) are both registered by Gentec Solo PE-2M (Lake Oswego, OR, USA) optical power meters equipped with UP19K-110F-H9 pyroelectric power detectors (Lake Oswego, OR, USA). The uncertainty of the power meter calibration is ±2.5%. Section 2.3 discusses the real-time control of plasmonic structure fabrication applied here.

### 2.3. Real-Time Control: Background

**Figure 3:**
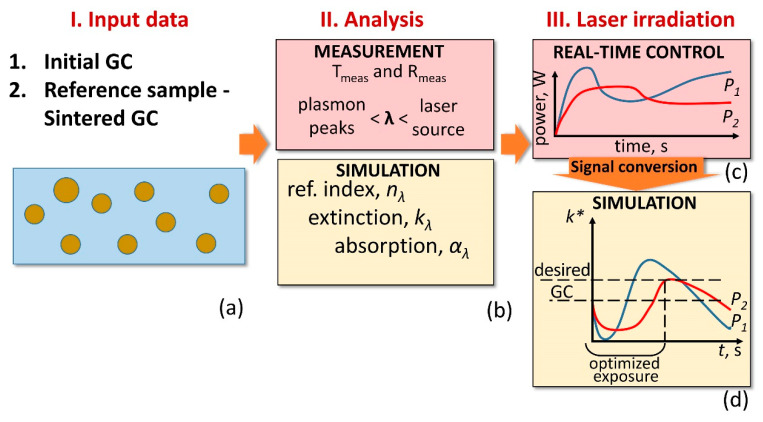
Schematics of the optimization procedure used to fabricate structures with desired optical properties inside GC: (**a**) input data as the sample to irradiate and the reference one—SGC; (**b**) measurement and simulation of the samples optical characteristics; (**c**) real-time monitoring of transmitted laser power; and (**d**) mathematical conversion of the registered laser power to extinction (k*) behaviour at a specific period of the irradiation time.

The proposed real-time control procedure aims to optimize the laser parameters of GC irradiation by providing the laser power feedback for fabricating the buried microstructure with desirable spectral characteristics. Figure 3 schematically shows the procedure and it involves the following steps.

(i) First, we set input data: the material to irradiate – GC, and the reference sample, which possesses the desired optical properties of the structure to fabricate, in our case it is SGC (Figure 3a).
(ii) Subsequently, the transmittance and reflectance of the samples are measured (Figure 3b). The registration range covers possible plasmonic peaks and applied laser wavelength. Simultaneously, the main optical constants, namely, the absorption (*α_λ_*), extinction (*k_λ_*), and refractive index (*n_λ_*) are estimated by the mathematical simulation. The optical properties of the structure to fabricate are homologated with the reference sample.
(iii) Next, GC laser irradiation is accompanied by the incident and transmitted laser power monitoring (Figure 3c).
(iv) Since the structure is fabricated inside GC, the transmitted laser power brings the key optical parameter, such as an extinction (k*). This enables us to leap ahead to convert the transmitted power signal into k* behaviour at a specific period of the irradiation time through the mathematical simulation (Figure 3d). Laser irradiation activates photo-thermo-chemical mechanisms that dramatically deviate the extinction curve and confuses the exposure time. Hence, it is also important to describe the mechanisms that are involved for any new glass composite to irradiate.
(v) Afterwards, the user sets the desired extinction for the structure to fabricate. For example, in the experiment, we associate the desired extinction with one of SGC (*k_λ_*) estimated in the second step. The convergence of both extinction values—the initial and reference one—shows the required exposure time and laser power to fabricate a plasmonic structure.

After spectral investigation of the fabricated structure, it becomes possible to estimate the size, concentration, and chemical composition of the secondary phase across the structure through the simulation that is based on the effective medium theory [19,25]. It is worth noting that this investigation is performed without fragmentation of the structure. The details are presented in Section 3.4.

## 3. Results and Discussion

### 3.1. Results of Glass Composite Laser Processing

**Figure 4:**
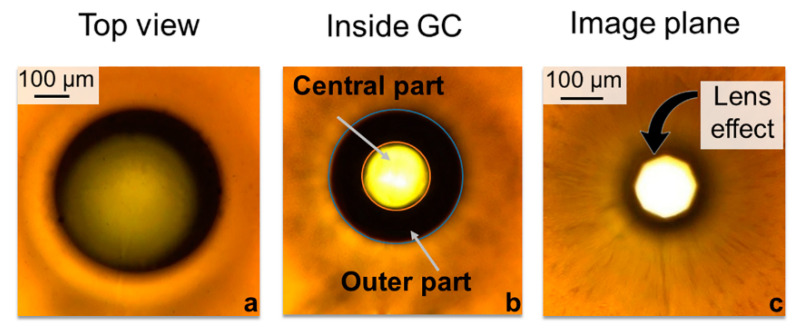
Laser-modified region fabricated at laser exposure of 15 s with power 13 W inside GC micro-photos captured in the transmission light at different position of the microscope image plane: (**a**) surface of modified region, (**b**) central part, and (**c**) under the region in its image plane.

Upon laser irradiation of GC, a modified region appears and it is clearly visible for power density in the range from 6.3 × 10^5^ to 1.9 × 10^6^ W/cm^2^. The formed regions are characterized by darkening in the exposure area under a microscope (Figure 4a). While displacing the image plane of the microscope, the modified region has the central part and the periphery (Figure 4b). The lateral size of the region is about 150 µm. The image indicates higher density in the central part than that in the non-irradiated region. It seems like a focused laser radiation forms a heat point source inside GC, which leads to the secondary phase redistribution across the irradiated region with the spaces in the central part [26] (Figure 4b). The subsequent shift of the image plane of the microscope shows the ability to recreate the image of the objects located on the optical axis. Since the diaphragm of the microscope lighting system is observed (Figure 4c), the fabricated structure plays the role of a microlens, where density is increased in the central part. Section 3.4 discusses the secondary phase determined by spectral investigation.

**Figure 5:**
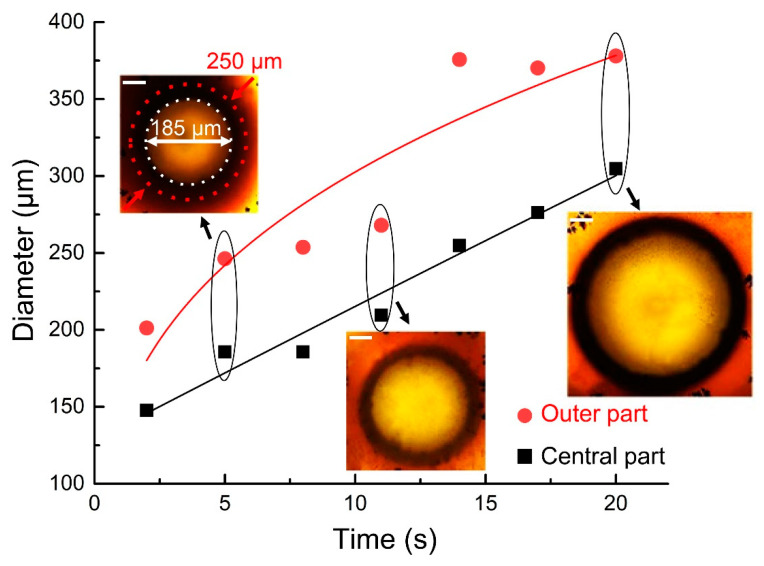
Time evolution of the fabricated structure centre and outer diameter in the process of 20 s laser exposure at *P_0_* = 9.6 W. Scale bar equals to 50 µm.

The size of the structure depends on laser power and exposure time. In the case of *P_0_* = 9.6 W, we experimentally observe that the diameter shrinks from 2 to 2.5 times during 15 s (Figure 5). Interestingly, over the next 5–10 s, the outer part size decreases by 1.5 times, i.e., it is practically restored. Consequently, all of the fabricated structures consist of two sections: optically transparent at the centre and obscured at the periphery.

### 3.2. Laser Power Monitoring and Mechanism Description

**Figure 6:**
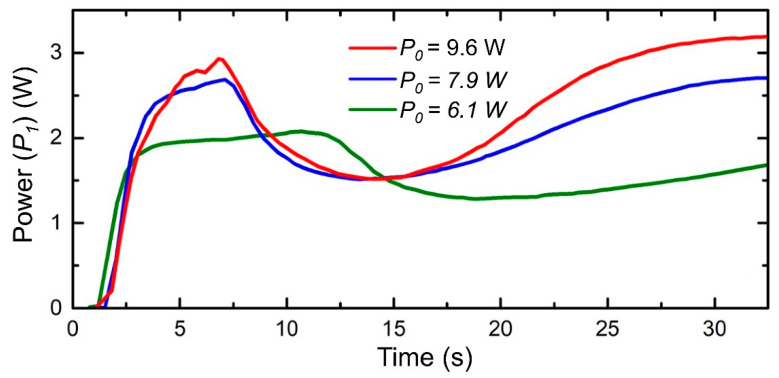
Time-evolution of the transmitted laser power (*P_1_*) during the structure fabrication, while the incident, *P_0_*, equals to 6.1 W (green), 7.9 W (blue), and 9.6 W (red).

The registration of the incident (*P_0_*) and transmitted (*P_1_*) laser powers shows the “breathing effect” in the transmitted power signal (Figure 6), which distinguishes three stages of the creation of the modified region. Stage I starts from the beginning of laser irradiation and captures an abrupt rise in the transmitted power for several seconds. Subsequently, transparency increases more slowly for 5–7 s. It continues for a longer time (12 s) for the lowest power (*P_0_* = 6.1 W). A sharp decrease in the transmission takes place during stage II. The GC darkening was clearly observed at this stage that lasts for 7–15 s and is shorter for larger laser powers. During stage III (after the delay of 16 s for *P_0_* = 7.9 W and 18 s for *P_0_* = 9.6 W) a partial transmission recovery is observed at laser power above 7 W. The recovery takes more time for lower laser powers.

We would like to remind that the final structure consists of several well-pronounced regions, as follows: the central part that is almost optically transparent and the heavily darkened periphery parts (Figure 4b). A better understanding of the mechanisms of such a structure formation is needed in order to understand the nature of the secondary phase redistribution generation and to determine the features homologated with the end of the structure fabrication. Generally, laser irradiation of a glass composite initiates a combination of the thermally activated nanoparticle growth and ion migration processes determining the optical properties of the composite material [27]. Albeit light absorption by metallic clusters is negligible for the considered laser wavelength (our laser operates at 1064 nm), our GC sample is locally heated inside, which is sufficient for material thermal expansion, sintering/densification, softening, etc. A photo-thermo-chemical mechanism is involved because the GC are photosensitive and the absorbed laser radiation heats the secondary phase.

Based on the laser power feedback, we identify the possible reasons for the GC optical properties changes, as follows: (i) halides decomposition to ions and water removal; (ii) a subsequent temperature increase leads to the nanoporous framework densification that also slows the ion migration outwards to the periphery; and, (iii) the ions thermal diffusion leads to nanoparticles growth around the laser-irradiated zone. In the central part, a small number of nanoparticles remains anyway, even with the laser source turned off, since the lifetime of the laser-induced thermal field is sufficient for thermal diffusion.

### 3.3. Simulation of Optical Properties

In the case of GC sintered in a furnace, the modification processes take place sequentially across the entire sample and they are determined by both temperature and exposure time. On the contrary, laser irradiation accelerates and localizes the processes in the interaction zone. The primary simulation of optical properties is performed for SGC, since the final optical properties of the structure to fabricate equal SGC. Pure FS and PG are applied as samples to compare. The absorption coefficient (*α_λ_*), extinction (*k_λ_*), and material refractive index (*n_λ_*) are derived from the measured *T_meas_* and *R_meas_* (Figure 1), while taking the samples thickness (*h*) into account [28]:

(1) $$T_{meas}=\frac{I_{T}}{I_{0}}=\frac{{\left(1-R_{\lambda }\right)}^{2}\exp\left(-\alpha_{\lambda }h\right)}{1-R_{\lambda }^{2}\exp\left(-2\alpha_{\lambda }h\right)}$$

(2) $$R_{meas}=\frac{I_{R}}{I_{0}}=R_{\lambda }[1+T_{meas}\exp\left(-\alpha_{\lambda }h\right)]$$

where *λ* is the laser radiation wavelength, *R_λ_* is inner reflection determined by the Fresnel equation [29] when considering the samples location in air:

(3) $$R_{\lambda }=\frac{{\left(n_{\lambda }+1\right)}^{2}+k_{\lambda }^{2}}{{\left(n_{\lambda }-1\right)}^{2}+k_{\lambda }^{2}}$$

**Figure 7:**
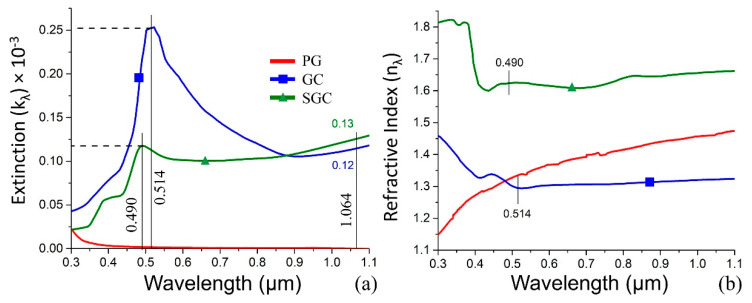
The simulation results: (**a**) the extinction, (**b**) refractive index for PG (red line), GC (blue line), and SGC (green line).

The absorption coefficient is calculated based on Equations (1) and (2), as follows:

(4) $$\alpha_{\lambda }=\frac{1}{h}\ln\left(L+\sqrt{L^{2}+R_{meas}^{2}}\right)$$

where *L* = (1 – *R_meas_*)^2^/(2*T_meas_*). Since *k_λ_* = *α_λ_λ/4π*, we estimate the extinction (Figure 7a). Currently, we suppose the internal and external reflection coefficients are such that the refractive index (*n_λ_*) can be evaluated, as follows (Figure 7b):

(5) $$n_{\lambda }=A+\sqrt{A^{2}-k_{\lambda }^{2}-1}$$

where *A* = (1 + *R_meas_*)/(1 – *R_meas_*).

The performed simulations show the peak wavelength and its shift from 514 nm to 490 nm after GC sintering (Figure 7). However, the extinction peak dramatically decreases (Figure 7a).

It is reasonable to suppose that the absorption of SGC is higher and the secondary phase volume was increased, which is also confirmed by the increased SGC refractive index (Figure 7b). The PG curve is presented here just to show the absence of any peak. The simulation shows that the extinction function is more informative when compared to the refractive index function. GC and SGC extinctions obtained for 1.064 µm allow for one to associate them as the bottom and upper limit for the structure to fabricate.

**Figure 8:**
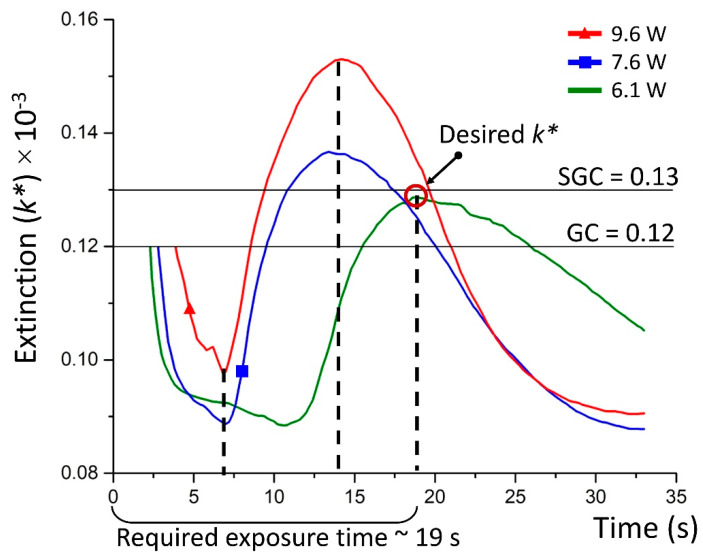
Simulated time dependent extinction (k*) changes during laser irradiation at different incident power: 9.6 W (red curve), 7.9 W (blue curve), and 6.1 W (green curve).

The calculations of the extinction coefficient are performed for the transmitted laser power to obtain the time dependent dynamic extinction change (k*) for the constant laser wavelength of l064 nm. Figure 8 demonstrated that the k* curves change as a function of the incident laser power. The previously estimated extinction coefficients for GC and SGC are also shown in the Figures by solid lines. The intersection of these curves corresponds to the material phase shift state changing from GC to SGC. In our case, the extinction of the fabricated structure has to converge the initial glass extinction and go to SGS one.

The k* dynamics is worth investigating (Figure 8). For the first 7 s we notice a sharp drop indicating the second phase, where halogenides dissipate in the irradiation region, i.e., silver bromide and iodide are decomposed. Subsequently, the temperature arises exceeding 400 °C, which enables free ions to collect in a nanoparticle. The nanoparticles growth provides greater absorption, which is seen in the curve increasing from 7 till 12 s. The increased absorption corresponds to the temperature increase until the critical value, preventing nanoparticles dissipation. Thus, k* decreases again, crossing SGC extinction state and then after a few seconds the GC state. All of this is true for the incident power equals 7.9 and 9.6 W. The opposite situation takes place for the smallest power 6.1 W, where the extinction goes to SGC one. For this, the laser exposure time equals to ~17 s. Thus, we obtain the desirable material state and there is no need for further laser irradiation. As a result, we suggest feedback based on the transmitted laser power to optimize the laser processing of GC.

We note that the main purpose of the laser source is to establish a sustainable heat source. The heat source activates the nanoparticles growth. Thus, the suggested procedure can be adopted for various types of laser irradiation, such as continuous radiation [30], as well as for a pulsed laser irradiation [31,32]. Another point is that a partial sample transmission is required to provide the registration of the transmitted laser power.

### 3.4. Plasmonic Properties and Nanoparticles Properties Simulation

**Figure 9:**
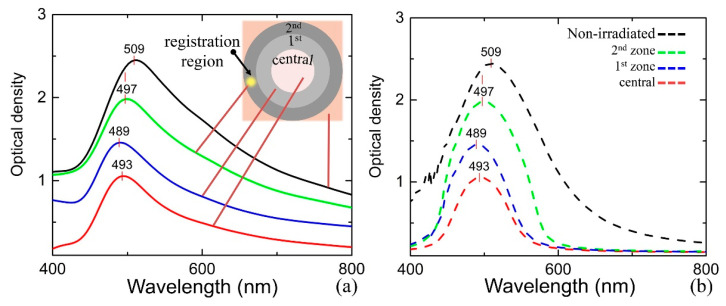
(**a**) Optical density spectra for the initial sample and measured at several distances from the centre of the fabricated structure. Inserted scheme represents the top view of the structure fabricated at 15 W and 20 s. (**b**) Simulation results providing the best fit to the experimental and yielding the mean nanoparticle diameter of 11 nm for the central part (optical density is peaked at 493 nm), 14 for the next ring (optical density is peaked at 489 nm), 30 nm (at 497 nm), and 4 nm for the outer region (at 509 nm).

Figure 9a shows the micro-spectroscopy results obtained for all zones of the fabricated structure. The presence of salt mixture in the nanoporous framework results in the plasmonic response appearance that is characterized by the absorption peak at *λ* = 509 nm. The obtained spectrum clearly indicates the presence of the secondary phase across the fabricated buried structure with different volume/concentration: central, 1st, and 2nd zones. The farther from its centre, the more pronounced and shifted the peak is. The larger is the nanoparticle size in the dielectric medium, the more the peak is shifted to the blue region. Spectral analysis across the structure shows a set of coaxial spheroids, where the outer is formed from the secondary phase with different-sized nanoparticles. Thus, secondary phase migration toward the periphery of the laser-affected volume is obvious.

**Figure 10:**
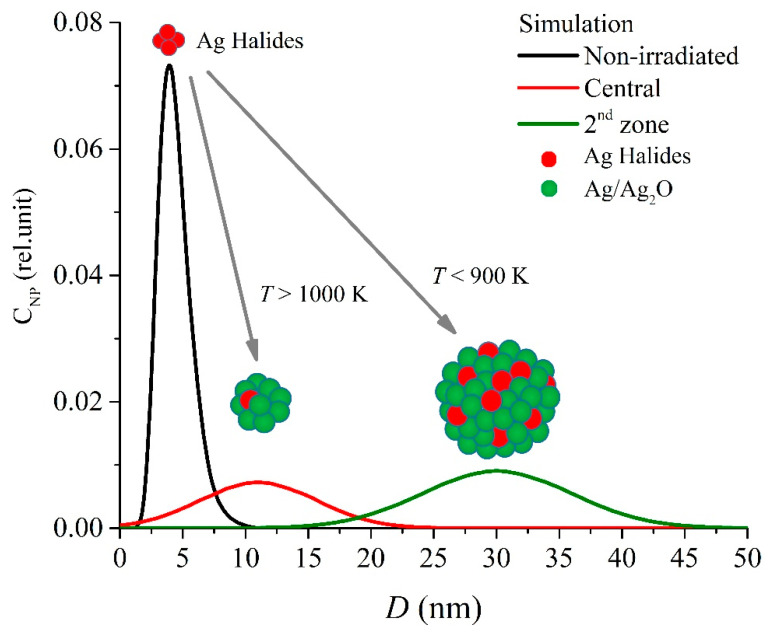
Simulated size (*D*) distribution of nanoparticles (concentration, *C_NP_*) in the sample for the non-irradiated zone (*C_NP_* = 0.073, *D* ~ 4 nm) and the fabricated structure: its central part (*C_NP_* = 0.0072, *D* ~ 10 nm) and the 2nd zone (*C_NP_* = 0.0090, *D* ~ 30 nm).

Based on the EMT [19,25], it becomes possible to simulate the spectra for each zone in the sample: central, the 1st one, the 2nd one, and the unirradiated one. A careful tuning of chemical composition, nanoparticles size, and metal volume ratio for effective mediums of each zone in the model allows for us to fit the measured spectra. The initial sample properties are determined based on the fitting of the following measurements [33]: dispersion of the pore size distribution, dispersion of nanoclusters distribution (Figure 10), mass fraction of the secondary phase, the initial concentration of the secondary phase atoms, and the spectral characteristics in the various parts of the sample. In the simulation, we consider the plasmon resonance from the nanoparticle assembly (Ag, AgCl, AgBr, AgI, Ag_2_O) distributed in a dielectric medium (Table 1). The initial composition of the secondary phase included silver halides in equal proportions according to the impregnation condition.

The following procedure of the concentration adjustment is then applied to simulate the nanocomposite optical properties. The GC consists of the framework dielectric function (*ε_m_*) and the secondary phase (*ε_ph_*), including its volume fraction (*ν_ph_*). The dielectric function of the effective medium holds true *ε_eff_* = (*n_λ_* + *i*k_λ_)^2^ = *f*(*ν_ph_*, *ε_ph_*, *ε_m_*). An approximation of isolated nanoparticles is used here. In this case, the distance between particles is larger than their size (*d >> 2r_Np_*). The Bruggeman approximation is chosen as the most suitable model presentation [19]. The complex GC description is accomplished by the Bergmann equation [25,33]. The thickness of the model sample equals 90 μm, corresponding to a photometric layer of the microscope-spectrometer.

The same procedure is performed for the laser-irradiated region, which consisted of the central part, the 1st, and the 2nd zones. However, the chemical composition of each zone depends on the behaviour of silver halides during laser-induced heating. Therefore, the following conditions are applied to tune the chemical composition for each zone (Table 1). In the central zone, when considering the sintering of the nanoporous matrix in the beam waist, which was discussed above, the transition temperature (up to 1000–1300 K) is achieved [34]. Thus, such silver halides are thermally decomposed, according to the handbook [35], as following: for AgI at 825 K, AgBr at 970 K, and AgCl at 1313 K. The decomposition leads to ions formation with following combination into nanoparticles. For the 1st and the 2nd zones, which are far from the beam waist, the temperature was lower. As a result, the most heat resistant silver halide (AgCl) remains practically unchanged. Thus, the initial nanoporous framework of these zones is filled with silver nanoparticles, which diffused from the central part, and with the silver halides (Table 1).

The optical density registration for each zone (Figure 9a) confirms the above discussion. Unlike silver nanoparticles, its oxide and halides do not have a pronounced plasmon resonance in the optical visible wavelength range. The silver oxide resembles to a semiconductor in view of its electronic structure and optical properties [36]. In fact, properties, such as concentration (*N*), effective mass (*m*), and relaxation time (*τ*), are smaller for silver halides and its oxide when compared to the pure silver (Table 2). The wavelength peak shifts to the IR region as well as its intensity decreases as a result of an increase in the fraction of silver halides. Thus, the peak position and its intensity are the reference characteristic for the estimation of the chemical composition of the secondary phase. The chemical composition (Table 1) in the model is corrected to fit the simulated spectrum to the measured one in the spectral range 0.3–1.1 μm (Figure 9b). We can also see the plasmon resonance width in the simulated spectra, which turned out to be smaller when compared to the experimental data. Such a difference is probably due to the light scattering on the nanoporous framework, which is disregarded in the present model.

The nanocluster concentration is calculated as a volume ratio of the secondary phase by using the following distance-depending parameters: 0.41% in the initial PG, 0.1% in the central part of the laser-modified region, and 0.38% in the surrounding part. Thus, nanoparticle diameter is found to range from 11 to 30 nm inside and around the laser-affected zone, whereas this size is as small as 4 nm in the unmodified PG plate part.

The calculations of the spectral characteristics are based on the effective dielectric function, *ε_eff_* [34]. This function accounts for the dielectric properties of the secondary phase in the form of silver nanoparticles, silver oxide, and halides in different proportions, as well as the host medium. The dielectric function of the secondary phase can be expressed in terms of the incident radiation, λ [37,38]:

(6) $$\varepsilon_{ph}\left(\lambda \right)=\varepsilon_{Bulk}\left(\lambda \right)+\varepsilon_{Shape}\left(\lambda \right)+\varepsilon_{Plasm}\left(\lambda \right)$$

The first term describes the properties of the bulk material:

(7) $$\varepsilon_{Bulk}\left(\lambda \right)=\sum_{i=1}^{I}\mu_{i}\varepsilon_{Com}{\left(\lambda \right)}_{i}$$

where *μ_i_* is the mass fraction of the components in the composition of nanoparticles. The values of ε_Com_(*λ*) for all components are taken from Ref. [39].

The second term accounts for the electronic properties of the nanoparticles with radius *r_NP_*

(8) $$\varepsilon_{Shape}\left(\lambda \right)=\frac{e^{2}\lambda r_{NP}}{2\pi c\varepsilon_{0}}\sum_{i=1}^{I}\frac{\mu_{i}n_{i}\lambda }{m_{i}\upsilon_{F,i}\left(2\pi c\mu_{i}\tau_{i}+i\lambda \right)}$$

where *r_NP_* = 0.5·D, *e* is the elementary charge, *c* is the speed of light in vacuum, *ε_0_* is the dielectric constant, and *n*, *m*, *τ*, *υ_F_* are the concentration, effective mass, relaxation time, and velocity near the Fermi level for free electrons, respectively. Table 2 presents the parameters used in the simulation. The determined *ε_eff_* allows for us to recover the transmission spectra (*T_s_*) by Equation (1), where absorption coefficient is used as *α = (4π/λ)Im((ε_eff_*)^0.5^). The final optical density plot is simulated as *OD* = lg(1/*T_s_*) (Figure 9b).

## 4. Conclusions

In conclusion, we have developed and described a novel real-time control procedure combining both experimental measurements and simulation to fabricate micro-sized structures inside GC. In particular, we have simulated the time dependent changes in the optical properties of the laser-irradiated nanoporous glass composite. In the proposed procedure, only the experimentally obtained transmitted laser power and numerically calculated time-dependent optical properties are used in order to optimize laser irradiation parameters. Subsequently, the optical non-contact methods have been applied to investigate the fabricated structure. The EMT is used to connect the measured spectrum from each layer of the fabricated region and to estimate size, concentration, and chemical composition of the secondary phase across the layer. Every new glass composite to irradiate requires the mechanism interpretation as an integral part of the procedure. All of the involved steps make the procedure suitable for an effective monitoring of the plasmonic properties of the fabricated structures.

In addition, a curious “breathing effect” of transmittance has been demonstrated and explained. On one hand, the measured time-evolutions of the transmitted laser power correlate to the transitional material changes, such as softening, pore shrinking, dilatation, and cavitation. As a result, the porous structure is firstly erased and then renewed in the laser-modified volume. Shrinking pores prevent metallic species escape, while opening pores allow this process again. On the other hand, these results are also affected by the evolution of bimetallic nanoparticles that grow mostly at the periphery of the hot region at moderate laser heating. The final laser-induced structure, being strongly dependent on laser power and laser irradiation time, has been represented by a set of coaxial spheroids. If laser energy is high enough, the outer spheroidal shell is mostly composed of the secondary phase. In addition to the phase transitions and pore renewal, the formation of the observed microstructure has been attributed to nanocluster formation and metallic species migration that arise from the laser-generated temperature field.

The fabrication of such buried microstructures in a glass composite is particularly promising for numerous photonic and plasmonic applications.

## Figures and Tables

**Table 1:** Chemical composition of the secondary phase in the nanoporous framework before and after laser irradiation.

Zone	Cu	Cu_2_O	Ag	Ag_2_O	AgCl	AgBr	AgI	N_2_	O_2_	Ar	H_2_O
Initial GC											
Non-irradiated	1	2	1	0	32	32	32	64	17.2	0.8	18
Fabricated structure											
Center	1	2	83	14	0	0	0	78.1	21	0.9	0
1st			32	20	25	15	5				
2nd			16	6	30	30	15				

**Table 2:** Values of parameters for different components of the secondary phase.

	Cu	Cu_2_O	Ag	Ag_2_O	AgCl	AgBr	AgI
*N* (cm^−3^) *m* *τ* (s) *υ_F_* (m/s)	8.47 × 10^22^ 1.49 8.52 × 10^−15^ 1.287 × 10^6^	5.47 × 10^22^ 0.98 1.18 × 10^−14^ 1.466 × 10^6^	5.86 × 10^22^ 0.55 9.20 × 10^−15^ 1.876 × 10^6^	2.86 × 10^22^ 0.7 1.10 × 10^−14^ 1.568 × 10^6^	2.59 × 10^19^ 0.25 1.0 × 10^−4^ 2.685 × 10^6^	1.85 × 10^18^ 0.177 5.0 × 10^−8^ 2.924 × 10^6^	1.4 × 10^20^ 0.147 1.0 × 10^−7^ 3.312 × 10^6^
